# Mimickers of breast malignancy: imaging findings, pathologic concordance and clinical management

**DOI:** 10.1186/s13244-021-00991-x

**Published:** 2021-04-20

**Authors:** Mary S. Guirguis, Beatriz Adrada, Lumarie Santiago, Rosalind Candelaria, Elsa Arribas

**Affiliations:** Breast Imaging Department, MD Anderson Cancer Center, 1515 Holcombe Blvd., Unit 1350, Houston, TX 77030-4009 USA

**Keywords:** Radiologic-pathologic concordance, Breast cancer, Benign breast disease, Benign breast masses, Inflammatory breast disease

## Abstract

Many benign breast entities have a clinical and imaging presentation that can mimic breast cancer. The purpose of this review is to illustrate the wide spectrum of imaging features that can be associated with benign breast diseases with an emphasis on the suspicious imaging findings associated with these benign conditions that can mimic cancer. As radiologic-pathologic correlation can be particularly challenging in these cases, the radiologist’s familiarity with these benign entities and their imaging features is essential to ensure that a benign pathology result is accepted as concordant when appropriate and that a suitable management plan is formulated.

## Keypoints

- A heterogeneous group of benign breast conditions can mimic breast cancer.
- Understanding the imaging spectrum of benign breast diseases ensures appropriate radiologic–pathologic correlation.
- Appropriate radiologic–pathologic correlation is essential to avoid delay in proper management.

## Background

The clinical presentation of several benign breast conditions, common and rare, can mimic breast cancer. Suspicious imaging features may be part of the imaging spectrum of many benign breast conditions, making them indistinguishable from breast cancer. Although biopsy is often required to confirm the diagnosis, understanding the range of clinical and imaging findings is important to ensure appropriate radiologic-pathologic correlation and clinical management.

**Fig. 1:**
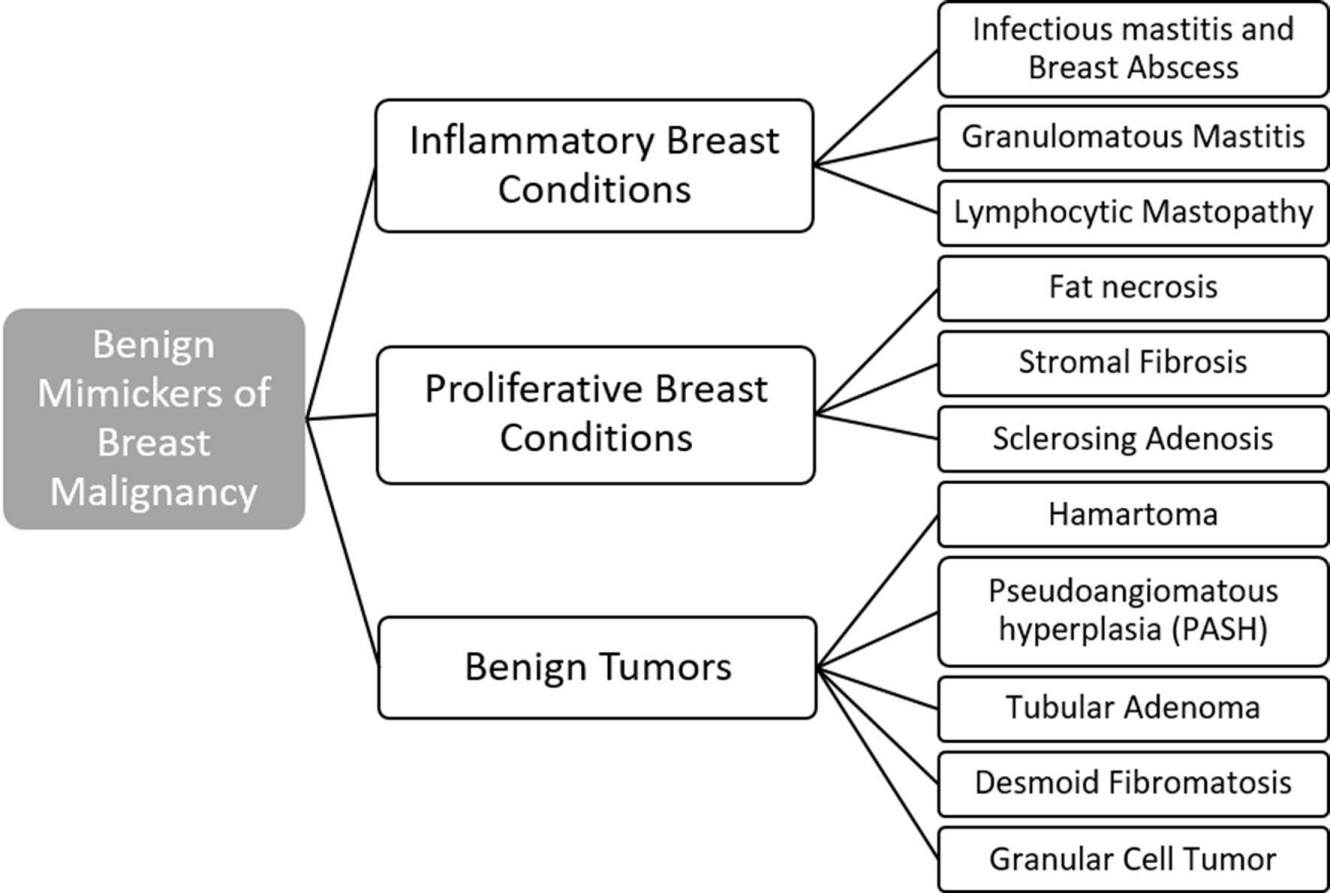
Categories of benign breast diseases that can mimic breast cancer

We have classified mimickers of breast cancer into three groups: inflammatory breast conditions, proliferative breast conditions, and benign breast tumors (Fig. 1). Benign inflammatory breast conditions that mimic malignancy include infectious mastitis and breast abscess, granulomatous mastitis, and lymphocytic mastopathy. Proliferative breast conditions that mimic malignancy include fat necrosis, stromal fibrosis, and sclerosing adenosis. Benign tumors that mimic malignancy include hamartoma, pseudoangiomatous hyperplasia, tubular adenoma, desmoid fibromatosis, and granular cell tumor. The purpose of this review is to illustrate the wide range of suspicious mammographic, sonographic, and magnetic resonance imaging (MRI) features associated with benign breast diseases. Recognition of these conditions is essential to ensuring careful and accurate radiologic-pathologic correlation, and to formulating a clinical management plan.

## Inflammatory breast conditions

Benign inflammatory breast conditions constitute a heterogeneous group of breast conditions characterized by a marked inflammatory process. These conditions are clinically important because they closely mimic and are often clinically and radiologically indistinguishable from inflammatory breast cancer [1]. Thorough imaging assessment of these cases is important. Biopsy is usually indicated to establish the correct diagnosis and to rule out breast cancer. Less-common inflammatory conditions that will not be described here include those associated with connective tissue disorders such as Churg–Strauss syndrome, amyloidosis, granulomatosis with polyangiitis (formerly known as Wegener’s granulomatosis), and sarcoidosis.

### Infectious mastitis and breast abscess

Breast abscess is a complication of infectious mastitis. Abscesses can be associated with lactation, in the case of puerperal abscesses, or independent of pregnancy, in the case of nonpuerperal abscesses [2]. Puerperal abscesses tend to be peripheral in location and are often easily recognized clinically. Nonpuerperal abscesses can pose a diagnostic challenge and are more commonly seen in younger women. They are usually periareolar and typically have worse outcomes and a higher rate of recurrence than puerperal abscesses. The risk factors for nonpuerperal breast abscesses are thought to include smoking and diabetes [3, 4].

**Fig. 2:**
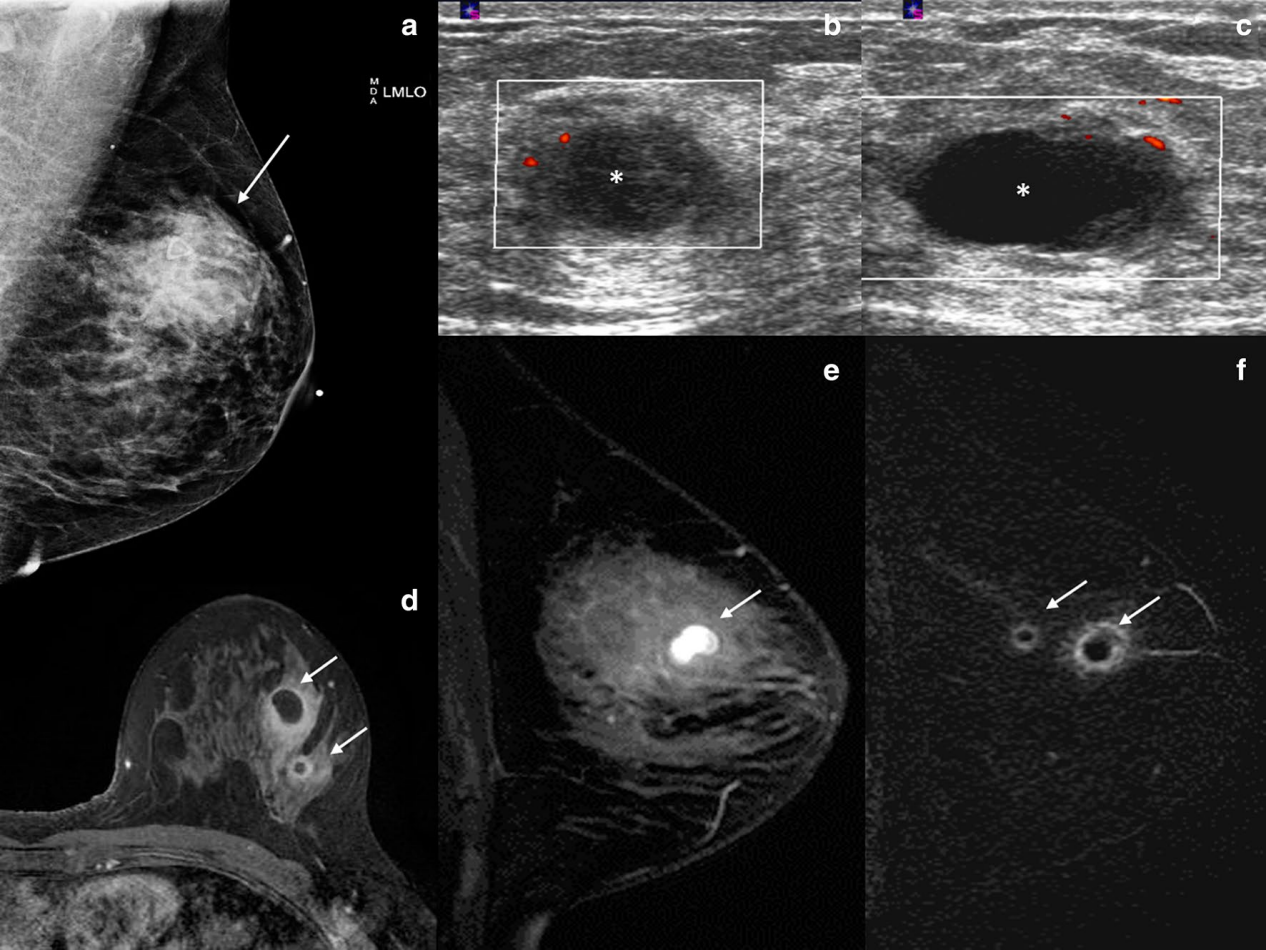
Breast abscess. A 45-year-old woman presented with a palpable area in the right breast. Mediolateral oblique (**a**) mammogram shows a focal asymmetry in the upper outer breast (arrow) and associated trinagular palpable marker. Power Doppler ultrasound images (**b**, **c**) reveal two hypoechoic, oval masses with peripheral vascularity in the same region (asterisks). Axial post-contrast T1-weighted MRI (**d**), sagittal T2-weighted MRI (**e**), and postcontrast subtraction T1-weighted MRI (**f**) show two T2-hyperintense, rim-enhancing masses (arrows). Needle biopsy showed acute inflammatory cells consistent with abscess without evidence of malignancy

Mammographically, mastitis and breast abscess present with skin thickening, asymmetry, a mass, or architectural distortion (Fig. 2a) [2]. Sonographic features of breast abscesses include one or more hypoechoic collections of variable shapes and sizes that are often continuous and multiloculated (Fig. 2b, c). Breast abscesses typically demonstrate a thick echogenic rim and increased vascularity, suggesting malignancy [2]. Associated mastitis presents as an area of increased parenchymal echogenicity, representing inflamed glandular parenchyma. Skin thickening, distended lymphatic vessels, and inflammatory axillary adenopathy can also be seen. On MRI, breast abscesses will typically be T2-hyperintense, have progressive enhancement kinetics, and sometimes have the characteristic thin rim of peripheral enhancement (Fig. 2d–f) [2].

As inflammatory breast cancer is the most important differential consideration with this clinical presentation, caution must be exercised to exclude an underlying malignancy. Patients with a clinical presentation typical of a breast abscess require a short-term, 7- to 14-day follow-up after treatment with antibiotics and drainage [2]. Inflammatory breast cancer should be strongly considered in patients with breast erythema and swelling in the absence of an abscess on ultrasound evaluation, especially in older non-lactating patients and in patients who are at increased risk of breast cancer. Mammography is indicated in these patients and should not be delayed. In lactating patients, although mammography is initially delayed until the acute symptoms of mastitis resolve following a course of antibiotics, mammography is indicated when there is a clinical suspicion for malignancy and in those with a prolonged clinical course [5, 6].

Although mastitis and breast abscess can be difficult to distinguish from inflammatory breast cancer, a number of imaging features tend to differ between them. While the skin thickening associated with inflammatory breast cancer is likely to be diffuse, the thickening associated with breast abscess and mastitis tends to be localized to the area involved with mastitis. A study by Chow found that suspicious microcalcifications are the most specific finding for breast cancer in patients with inflammatory breast symptoms of unclear etiology [7]. Nguyen et al. suggested that a mass with a hypoechoic wall and associated interstitial fluid is more suggestive of a breast abscess and not usually seen in the setting of breast cancer [8]. In addition, ultrasound evaluation of the axillary lymph nodes, in cases where malignancy is the primary consideration, is more likely to show markedly abnormal lymph node enlargement with the characteristic cortical thickening and hilar displacement of metastatic lymph nodes. In contrast, breast abscesses are more likely to be associated with reactive lymphadenopathy characterized by mild diffuse cortical thickening [2]. MRI can sometimes be used to differentiate the two entities: inflammatory breast cancer is more likely to show heterogenous enhancement with washout kinetics, while breast abscess is more likely to have an increased T2 signal and benign enhancement kinetics [2]. In patients with a prolonged course and patients whose condition does not respond to antibiotics, breast biopsy is indicated and should not be delayed.

### Granulomatous mastitis

Granulomatous mastitis is an inflammatory breast condition of unknown etiology. Pathologically, it is characterized by a non-caseating granulomatous inflammatory process of the breast lobules without an identifiable infectious or inflammatory etiology [9, 10]. Fat necrosis, abscess formation, and fibrosis are commonly associated end-stage features of this disease process [1]. Granulomatous mastitis is a rare diagnosis of exclusion. An inflammatory or infectious etiology such as plasma cell mastitis, granulomatosis with polyangiitis, sarcoidosis, or tuberculous mastitis must be excluded [11]. These conditions are usually distinguishable histologically. Granulomatous mastitis typically affects parous women of childbearing age, often within 6 months of pregnancy, although the timing of onset after pregnancy can vary widely and has been reported to be as long as 9 years post-partum in some cases [10]. Granulomatous mastitis typically presents as a firm palpable mass that is sometimes associated with skin erythema or pain [11]. Other symptoms include draining sinus tracts and nipple discharge.

**Fig. 3:**
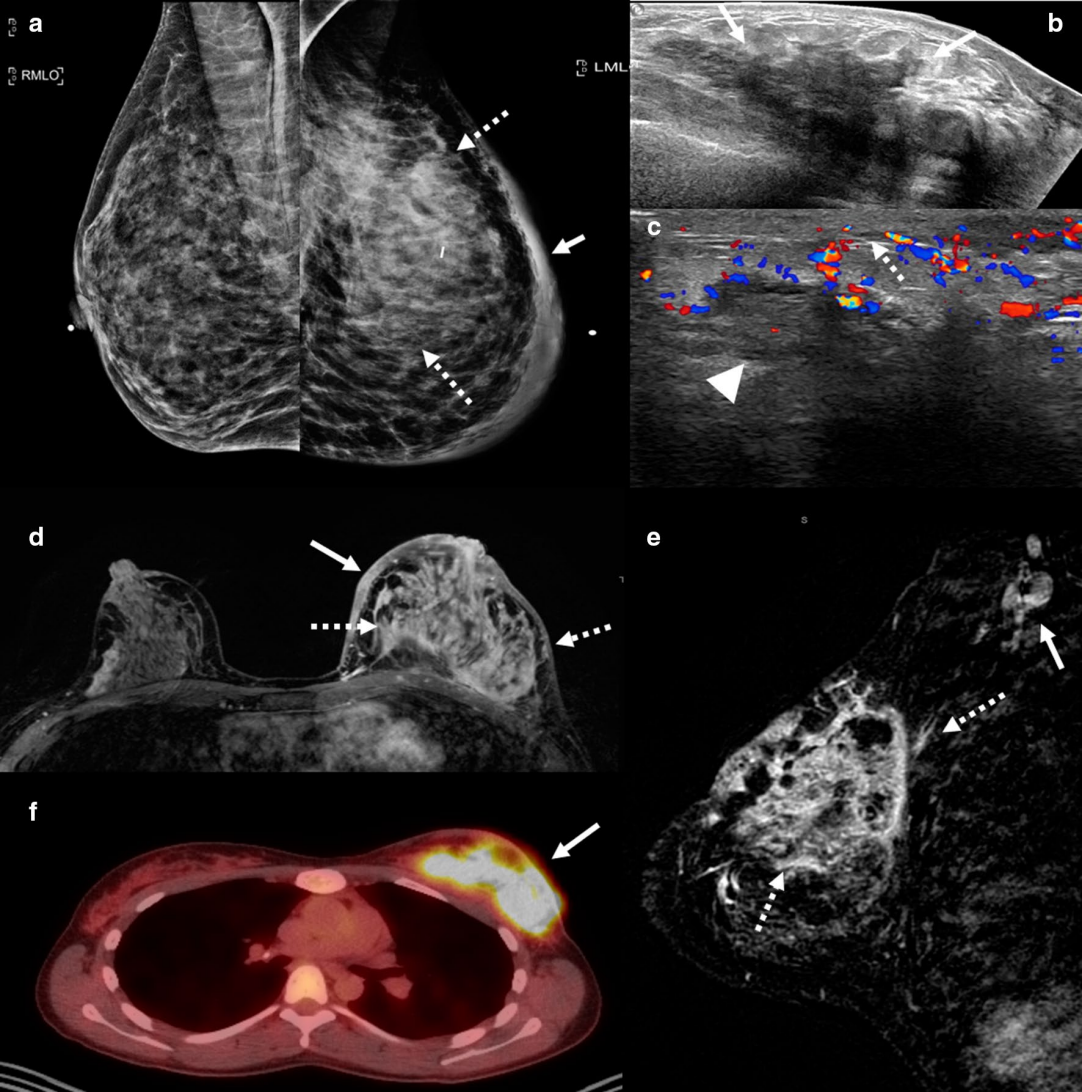
Granulomatous mastitis. A 34-year-old woman who is 2 years post-partum presented with a palpable left breast mass, diffuse breast swelling, tenderness, and erythema for several weeks. Bilateral mediolateral oblique mammogram (**a**) show diffuse skin thickening (solid arrow), global asymmetry, and trabecular thickening (dashed arrows) in the left breast, asymmetric from the right breast. Extended field of view grayscale ultrasound (**b**) shows an ill-defined hypoechoic, irregular mass (arrows). Color Doppler ultrasound (**c**) shows increased vascularity, edema (arrow head), and skin thickening (dashed arrow). T1weighted axial delayed post-contrast (**d**) and sagittal subtraction (**e**) post-contrast MRI shows diffuse skin thickening (solid arrow) and trabecular thickening with heterogenous enhancement involving the left superior breast (dashed arrows). There is associated axillary adenopathy (solid arrow in **e**). Axial PET/CT (**f**) shows diffuse fluorodeoxyglucose avidity involving the left breast (arrow). Findings and clinical presentation were suspicious for inflammatory breast cancer. The patient underwent three core needle breast biopsies of the mass in the left breast over the span of a month, and a skin punch biopsy. All biopsies showed dense stromal fibrosis, chronic inflammation, and features suggestive of granulomatous mastitis without atypia or malignancy

Mammographically, granulomatous mastitis can have a variety of presenting features such as masses, asymmetries, or trabecular and skin thickening (Fig. 3a). In some cases, the findings are mammographically occult. Sonographically, one or more commonly multiple irregular hypoechoic masses have been described (Fig. 3b) [11–14]. These masses can sometimes be continuous and can appear tubular. In other cases, ultrasound shows only parenchymal distortion with increased shadowing, without a discrete mass (Fig. 3c) [11]. Associated skin thickening and edema have also been described [11]. On MRI, the two most common findings are masses with circumscribed margins and rim enhancement and heterogeneous non-mass enhancement in a segmental or regional distribution (Fig. 3d, e) [15]. Reactive lymphadenopathy may also be present (Fig. 3e). Most cases of granulomatous mastitis have benign persistent enhancement kinetics, although washout kinetics can also be seen, making MRI unreliable in distinguishing between inflammatory breast cancer and granulomatous mastitis [16–18]. Positron emission tomography (PET)/CT can show fluorodeoxyglucose avidity (Fig. 3f).

Because the clinical and radiological presentation of granulomatous mastitis is indistinguishable from breast cancer, core needle biopsy is indicated to establish the diagnosis. Once concordance of the biopsy results is established, granulomatous mastitis is traditionally treated with corticosteroids or surgical intervention, such as wide local excision of localized disease or rarely mastectomy when the disease is extensive and resistant to corticosteroid therapy [19]. In cases resistant to corticosteroids, methotrexate can be used. In recent years, expectant conservative management is becoming the treatment of choice, especially in mild cases. Spontaneous resolution has been reported in about half of patients [20]. The prognosis of granulomatous mastitis is favorable, although some cases can be refractory to therapy and can develop significant sinus tracts and scarring [11].

### Lymphocytic mastopathy

Lymphocytic mastopathy is a perivascular and perilobular inflammatory process of the breast parenchyma incited by the infiltration of lymphocytes [21–24]. Clinically, it presents as one or more painless, mobile, discrete breast masses, closely mimicking malignancy. Multicentric and bilateral involvement is frequent. In most cases, lymphocytic mastopathy is associated with diabetes; however, other autoimmune disorders such as Hashimoto’s thyroiditis, Sjögren’s syndrome, and systemic lupus erythematosus have also been associated with lymphocytic mastopathy [1, 21, 25, 26]. Lymphocytic mastopathy has also been described in the absence of an underlying systemic condition. When associated with diabetes, this entity is often referred to as diabetic mastopathy and is most often seen in patients with a long-standing history of insulin-dependent diabetes. It has also been described in men in association with gynecomastia [26, 27].

**Fig. 4:**
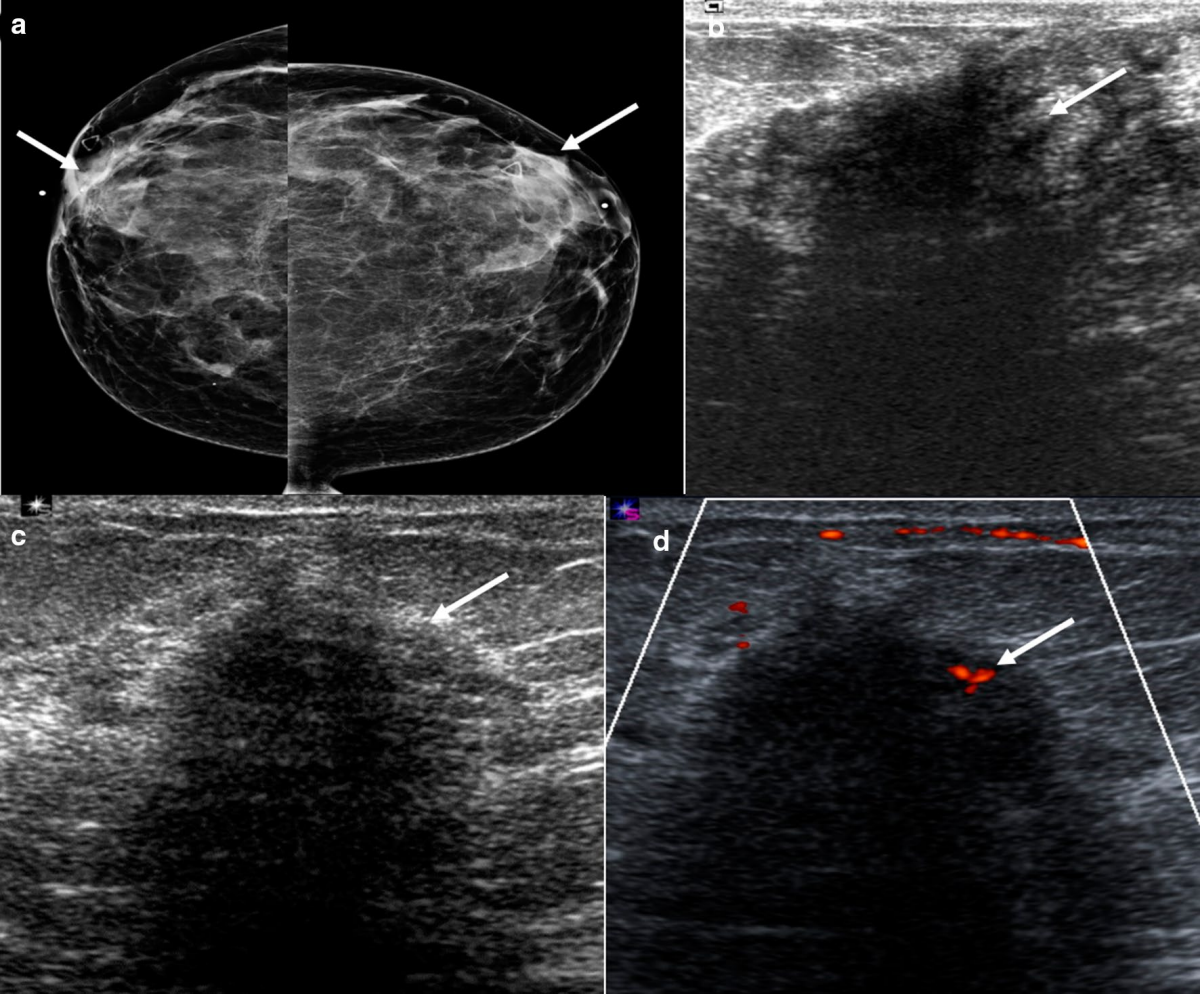
Diabetic mastopathy. A 56-year-old patient presented with bilateral palpable breast masses and an 8-year history of type 2 diabetes. Bilateral craniocaudal mammogram (**a**) shows bilateral non-calcified, obscured masses correlating with the palpable triangular markers (arrows). Grayscale right (**b**) and left (**c**) breast ultrasound shows irregular, hypoechoic masses with posterior acoustic shadowing (arrows). Power Doppler (**d**) ultrasound demonstrated internal vascularity (arrow) involving these masses. Core needle biopsy of both masses showed perilobular lymphocytic infiltration. Repeat core needle biopsy 8 months after the initial biopsy showed chronic lymphocytic lobulitis. No evidence of malignancy. Findings are consistent with diabetic mastopathy

**Fig. 5:**
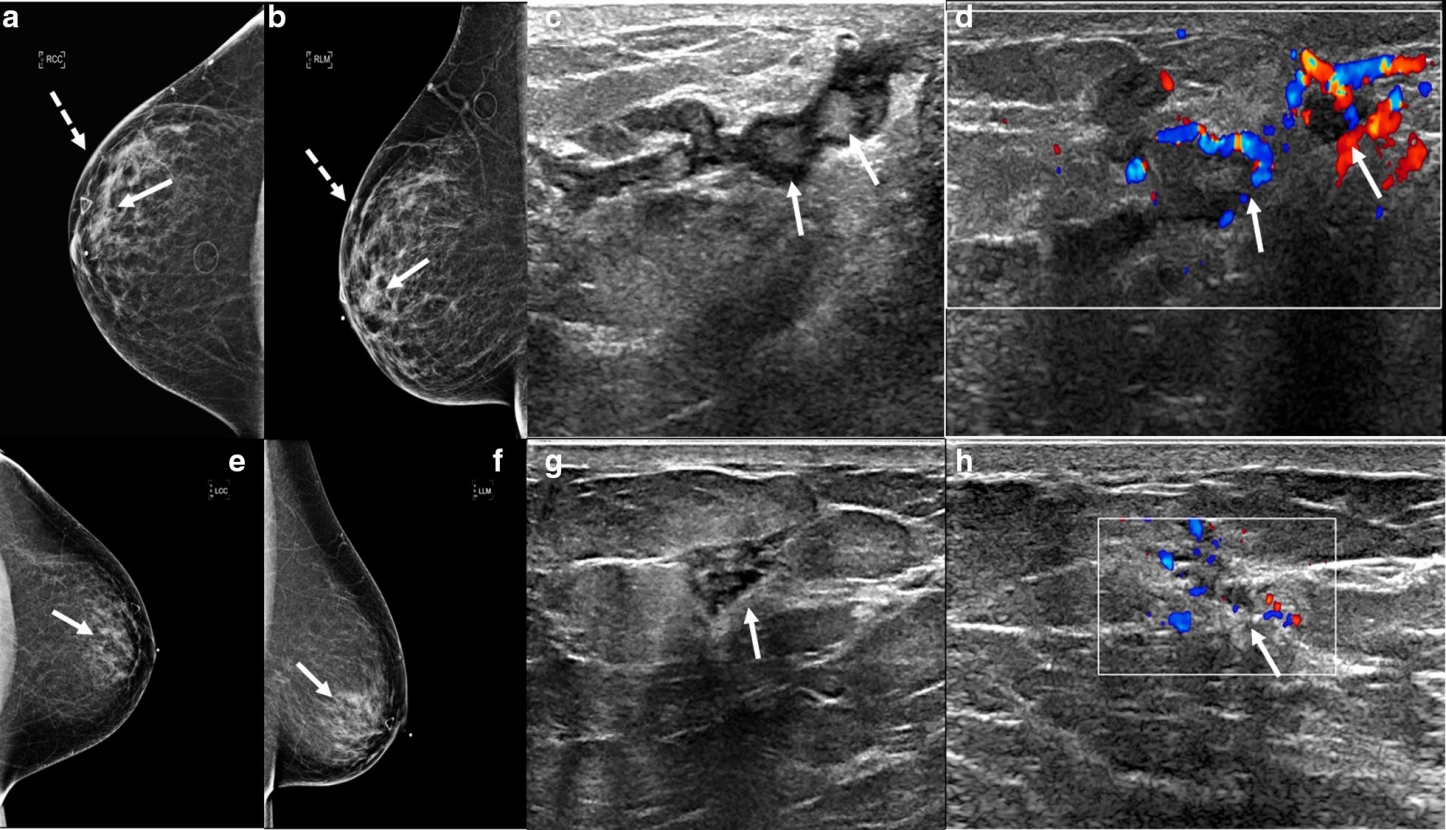
Lymphocytic mastopathy. A 37-year-old woman presented with right breast palpable retroareolar mass, swelling, erythema, and tenderness. Craniocaudal and lateral mammography (**a**, **b**) shows a focal asymmetry (solid arrows) and skin thickening (dashed arrows) associated with the triangular palpable marker in the lateral breast. Grayscale (**c**) and color Doppler breast ultrasound (**d**) reveals multiple retroareolar, vascular masses (arrows). Core needle biopsy showed acute and chronic inflammation and granulation tissue. The patient’s symptoms resolved within a month. The patient presented 2 years later with a contralateral palpable breast mass and nipple inversion. Craniocaudal and lateral mammographic views (**e**, **f**) reveal a focal asymmetry in the central breast associated with the palpable marker (arrows). Grayscale (**c**) and color Doppler (**d**) breast ultrasound reveals a vascular heterogeneous mass (arrows). Core needle biopsy revealed benign breast tissue with lymphoplasmacytic infiltrate. The patient’s symptoms improved over the following 6 months. She did not have a history of diabetes or other known immunologic condition

Mammographically, lymphocytic mastopathy presents as an ill-defined mass or asymmetry. Many times, it is mammographically occult. On sonography, it frequently presents as a hypoechoic irregular mass with posterior acoustic shadowing or an area of shadowing without a discrete mass (Fig. 4). Increased vascularity can also be seen (Fig. 5). MRI findings include heterogeneously enhancing masses and non-mass enhancement [28–30]. To date, there is no evidence that lymphocytic mastopathy is associated with an increased risk of developing lymphoma [22, 25]. The process is usually self-limited. Recurrence has been reported in patients who undergo surgical excision [21]. Therefore, close clinical and imaging follow-up is indicated to ensure that there is no interval development of new breast masses [22].

## Proliferative breast conditions

### Fat necrosis

**Fig. 6:**
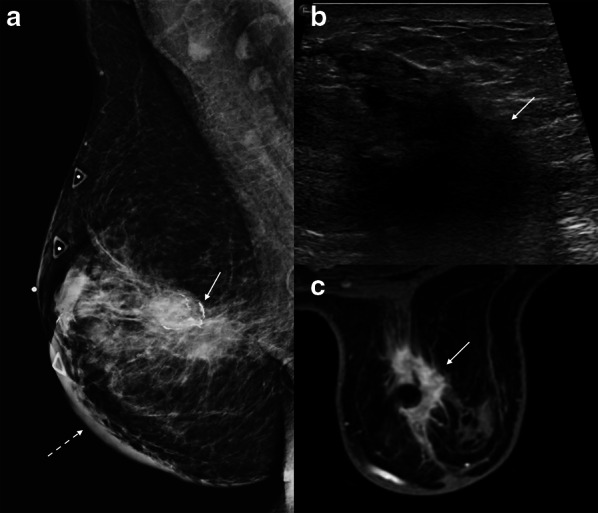
Fat necrosis. A 52-year-old woman with remote history of mastopexy presented with a palpable breast mass. Mediolateral oblique mammogram (**a**) shows an irregular mass with spiculated margins, associated coarse rim calcifications (solid arrow), and focal skin thickening (dashed arrow). Longitudinal grayscale ultrasound (**b**) shows a non-parallel irregular hypoechoic mass (arrow). Fat-suppressed axial post-contrast T1-weighted breast MRI (**c**) shows an irregular mass with spiculated margins (arrow) with associated singal void artifact related to post-biopsy clip marker. Ultrasound-guided biopsy showed fat necrosis. Repeat biopsy under MRI guidance confirmed the diagnosis of fat necrosis

Fat necrosis is a proliferative condition commonly seen as a result of breast surgery, infection, trauma, or radiation. Fat necrosis comprises 2.75% of breast lesions and is incited by the destruction of adipocytes, which causes an inflammatory process [31]. Clinical features of fat necrosis include palpable masses, pain, and skin changes such as skin tethering, skin thickening, and dimpling [32]. Fat necrosis has a wide spectrum of imaging features, some of which show the typical, classically benign findings, such as dystrophic calcifications or oil cysts. However, there are other imaging findings that can closely mimic malignancy: suspicious mammographic features of fat necrosis include irregular and spiculated masses, architectural distortion, asymmetries, coarse heterogeneous and even branching or pleomorphic calcifications. Ultrasound features of fat necrosis that mimic malignancy include irregular hypoechoic masses with posterior acoustic shadowing (Fig. 6). Characteristically benign features of fat necrosis on MRI include one or more fat-containing masses, which demonstrate T1 hyperintensity on non-fat supresed T1 sequences with corresponding drop in signal on fat-suppressed T1 and T2 sequences. Conversely, MRI features of fat necrosis that resemble malignancy are irregular enhancing masses, with or without a thick, irregular rim. Non-mass enhancement has also been reported [31]. If a PET/CT is performed, these lesions can be hypermetabolic [31].

The management of fat necrosis is based on the imaging features. Typical benign imaging features do not require further workup. Suspicious features including irregular masses, suspicious calcifications, and architectural distortions require image-guided biopsy. The radiologic-pathologic correlation of the suspicious imaging features of fat necrosis require the radiologist’s familiarity with the wide spectrum of suspicious imaging features of fat necrosis as well as careful review of the biopsy technique and confirmation of adequate sampling. Determining concordance can be challenging, especially on MRI-guided biopsies where the target may not remain visible and adequacy of sampling may be difficult to judge. If needed, a short-term follow-up MRI after biopsy may be recommended.

### Stromal fibrosis

**Fig. 7:**
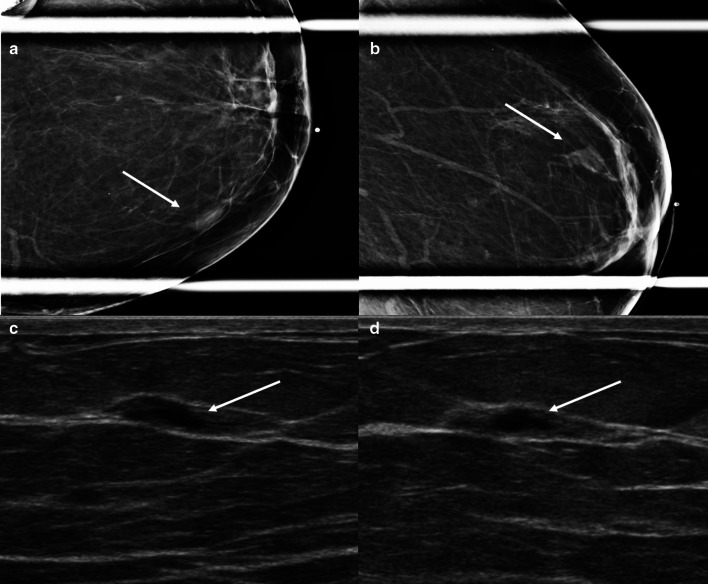
Stromal fibrosis. A 55-year-old woman presented for a focal asymmetry detected on screening mammography. Craniocaudal (**a**) and lateral (**b**) spot compression mammographic views show an oval non-calcified mass with angular margins (arrows). Grayscale transverse (**c**) and longitudinal (**d**) ultrasound show a mixed-echogenicity oval mass correlating with the mammographic finding (arrows). Ultrasound-guided biopsy yielded stromal fibrosis. Six-month follow-up mammography and ultrasound were recommended and demonstrated stability

**Fig. 8:**
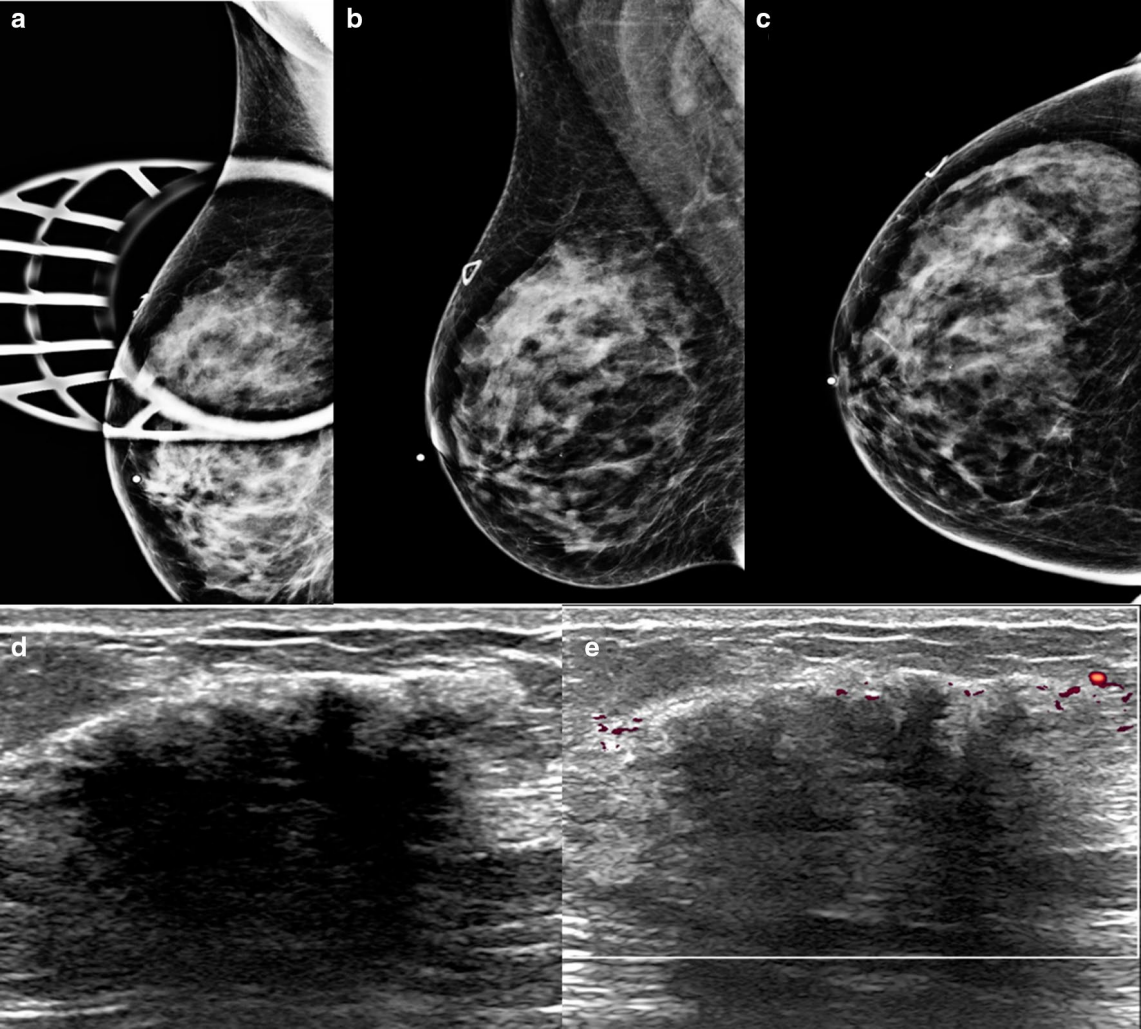
Stromal fibrosis. A 48-year-old female presented with a palpable breast mass. Spot compression tangential (**a**), mediolateral (**b**), and craniocaudal (**c**) mammographic views did not show a corresponding abnormality. Grayscale (**d**) and power Doppler (**f**) ultrasound images reveal an irregular mass with posterior acoustic shadowing corresponding to the palpable area. Core needle biopsy of the mass showed stromal fibrosis without evidence of malignancy

Stromal fibrosis in the breast is a benign pathologic entity characterized by proliferation of stroma with obliteration of the mammary acini and ducts on pathologic analysis. This process results in a localized area of fibrous tissue associated with hypoplastic mammary ducts and lobules. Stromal fibrosis is not an uncommon pathologic diagnosis, representing 2%-9% of biopsied breast lesions. This condition can manifest as a palpable mass or be clinically occult. The most common mammographic findings are calcifications, followed by masses and asymmetries (Fig. 7). With the advent of tomosynthesis, stromal fibrosis can present as architectural distortion. Sonographic findings of stromal fibrosis include masses and non-mass lesions. In most cases, the masses are oval or round, but stromal fibrosis can also present as irregular masses in 13% of cases (Fig. 8) [33]. MRI features include masses, non-mass enhancement and foci of enhancement [34]. The masses can demonstrate variable shapes and kinetics. It is important to note that stromal fibrosis can be seen in the setting of malignancy and has a reported upgrade rate to malignancy of 7% [35]. Therefore, evaluation of sampling adequacy and establishing radiologic-pathologic correlation in these cases is of utmost importance. Establishing a close relationship with the pathologist is essential to assess adequacy of the sample and whether repeat biopsy is indicated. Short-term follow-up is recommended to ensure stability [34–36].

### Sclerosing adenosis

**Fig. 9:**
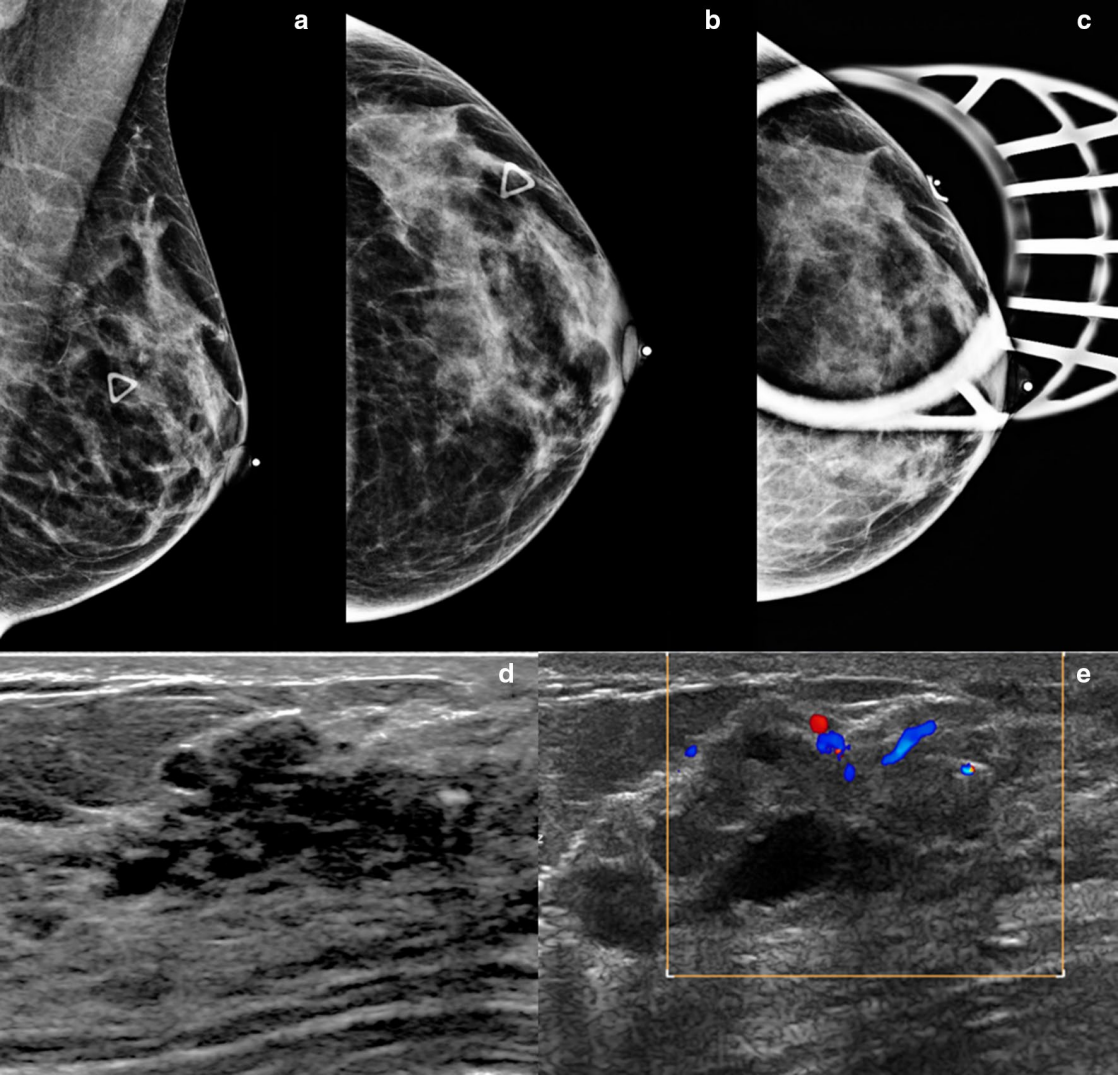
Sclerosing adenosis. A 35-year-old woman presented with a palpable breast mass. Mediolateral, craniocaudal, and spot tangential mammographic views do not reveal an abnormality (**a**–**c**, respectively). Grayscale and color Doppler ultrasound images (**d**, **e**) reveal a vascular, mixed cystic, and solid mass correlating with the area of palpable abnormality. Core needle biopsy showed sclerosing adenosis in a background of dense fibrosis without atypia or carcinoma. A 6-month follow-up ultrasound was rcommended and demonstrated stability

Sclerosing adenosis is a proliferative breast condition of the terminal lobular unit characterized by distortion of the lobules with an increased number of acini and desmoplasia. Sclerosing adenosis is often seen in perimenopausal women and can coexist with other benign proliferative lesions such as sclerosing papilloma, and complex sclerosing lesions [37]. Like stromal fibrosis, sclerosing adenosis can have features that resemble malignancy both clinically and radiologically. Additionally, this entity can be seen in the setting of malignancy [37]. Suspicious mammographic imaging features of sclerosing adenosis include amorphous, pleomorphic, and punctate calcifications. If a mass is seen as the presenting imaging feature, it can have irregular margins, although it more commonly has circumscribed margins. Architectural distortion can also be an imaging feature of sclerosing adenosis. Sonograpically, sclerosing adenosis can present as a circumscribed mass, with variable echogenicity. It can, however, mimic malignancy when presenting with suspicious features such as an irregular mass or focal areas of shadowing without a mass (Fig. 9).

The management of sclerosing adenosis ultimately relies once again on the imaging features. If the radiographic presentation is of a circumscribed mass or amorphous or punctate calcifications in a grouped distribution with adequate sampling, a pathologic diagnosis of sclerosing adenosis can be considered concordant. If the imaging findings are more suspicious such as an irregular mass or pleomorphic calcifications in a segmental or linear distribution, repeat biopsy is recommended to exclude a coexisting malignancy. If the sclerosing adenosis is associated with a radial scar, surgical excision is recommended. In cases of well-sampled architectural distortion, a short-term follow-up is recommended. A short-term follow-up is also recommended for circumscribed masses to ensure stability.

## Benign breast tumors

### Hamartoma

**Fig. 10:**
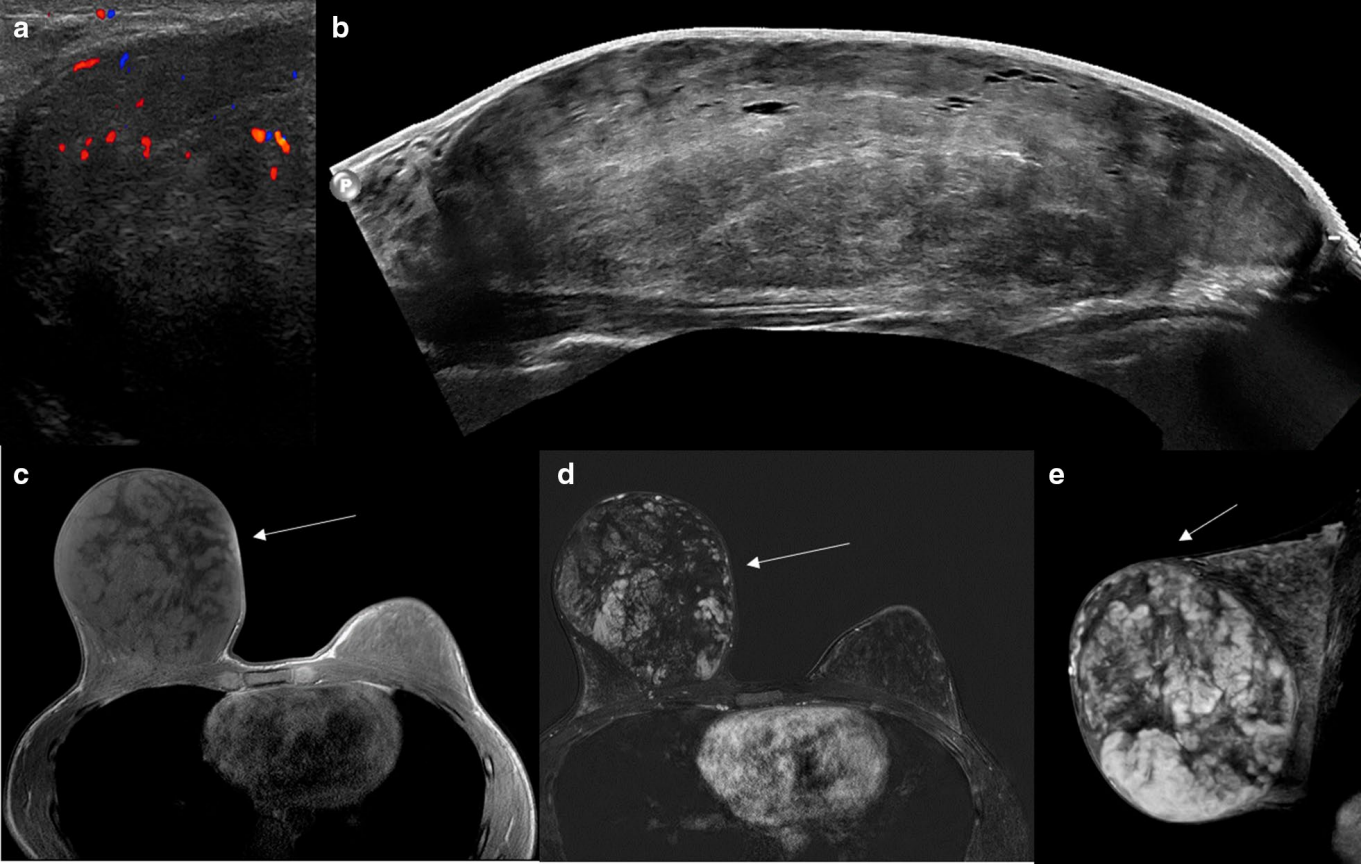
Hamartoma. A 14-year-old presented with unilateral breast enlargement. A mass was not palpable on examination. Color Doppler (**a**) and longitudinal panoramic ultrasound grayscale images (**b**) show a hypervascular, 17-cm isoechoic breast mass replacing the entire breast. T1-weighted non-fat-suppressed (**c**) and T1-weighted fat-suppressed (**d**) post-contrast MRI images show a well-circumscribed, heterogeneously enhancing breast mass suspicious for a sarcoma (arrow). T2-weighted sagittal images (**e**) show moderate T2 signal with marked heterogeneity (arrow). Pathology at the time of surgical excision showed hamartoma with areas of pseudoangiomatous stromal hyperplasia and fibroadenomatoid change

Hamartomas are uncommon, slow-growing tumors that represent 4.8% of benign breast tumors [38–40]. Often termed a “breast within a breast”, breast hamartomas are circumscribed benign tumors that are composed of variable amounts of fat, fibrous tissue, and glandular tissue. Mammographically, a hamartoma has a characteristic benign appearance of a fat-containing circumscribed mass. On ultrasound, a hamartoma presents as a parallel mass with circumscribed margins and heterogonous echotexture due to variable amounts of fat and glandular tissue. On MRI, hamartomas present as a heterogeneous circumscribed mass with a thick capsule, usually with heterogenous progressive enhancement kinetics [39]. Although most hamartomas have a typical benign appearance, they can mimic circumscribed malignant tumors, such as phyllodes tumor or breast sarcoma, if large or when presenting with marked heterogenous enhancement (Fig. 10) [41].

### Pseudoangiomatous stromal hyperplasia

Pseudoangiomatous stromal hyperplasia (PASH) is a benign breast entity that presents with a wide spectrum of imaging features. It is characterized by proliferation of the fibrous stroma lined by a complex network of slit-like spaces and slender spindle cells. PASH is associated with hormone exposure, including oral contraceptive use, and is primarily seen in premenopausal and perimenopausal women on hormone replacement therapy [42, 43]. It has been postulated that PASH may result from an abnormal reactivity of myofibroblasts to hormonal exposure, although the true etiology is still unclear [44, 45]. Histologically, the differential diagnosis includes a low-grade angiosarcoma and phyllodes tumor [46]. This pathologic entity can frequently coexist with both benign and malignant breast lesions. In one study, PASH was seen in up to 23% of biopsied cases which reflects its wide spectrum of imaging findings [47].

**Fig. 11:**
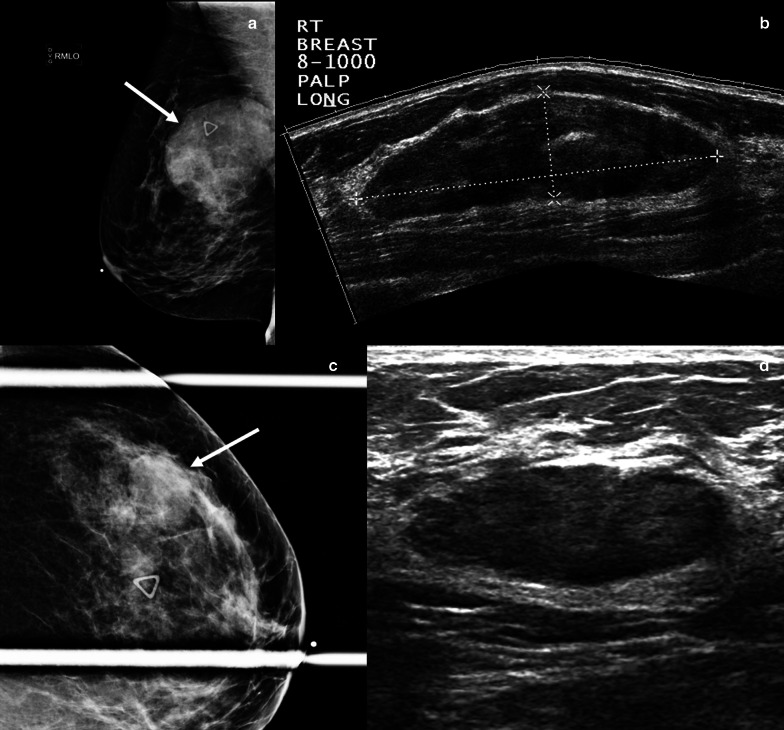
Pseudoangiomatous stromal hyperplasia. A 41-year-old woman presented with a palpable breast mass. Mediolateral oblique mammogram (**a**) shows a high-density well-circumscribed mass (arrow). Longitudinal grayscale ultrasound (**b**) shows a large, oval circumscribed mass. Core needle biopsy showed pseudoangiomatous stromal hyperplasia (PASH). A different patient, a 40-year-old woman, was found to have a new breast mass (arrow) on mammography, craniocaudal spot view (**c**). Transverse grayscale (**d**) ultrasound reveals a corresponding oval isoechoic circumscribed mass. Core needle biopsy showed PASH

**Fig. 12:**
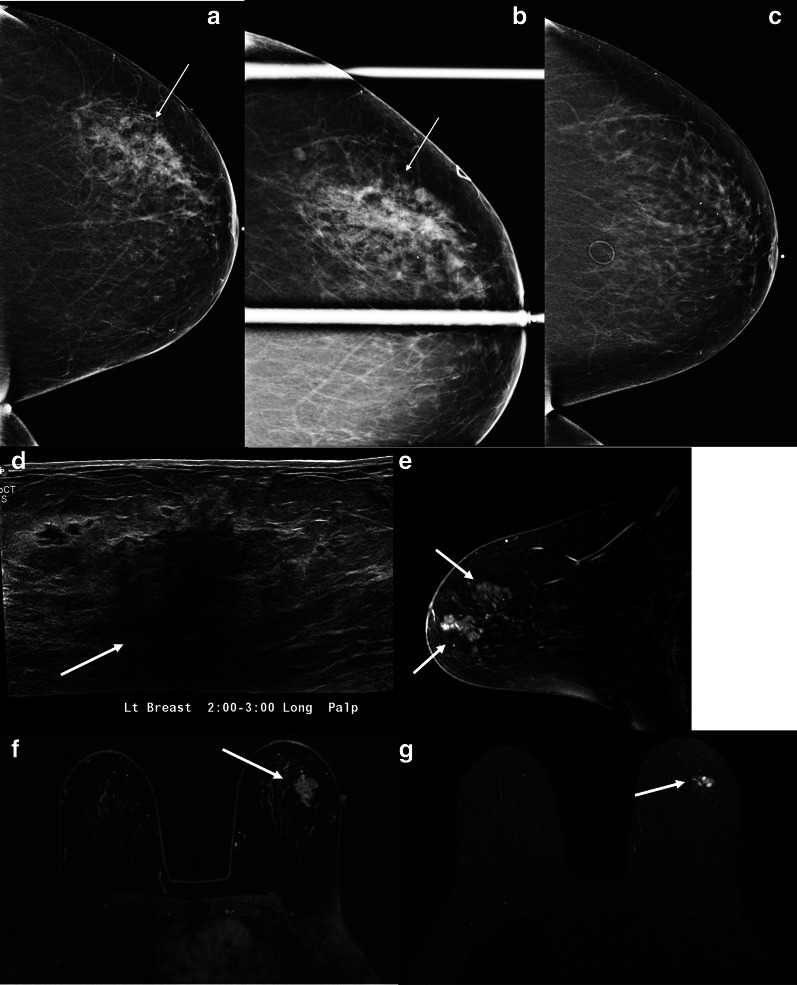
Suspicious presentation of pseudoangiomatous stromal hyperplasia. A 56-year-old woman with a new focal asymmetry in the outer breast that was identified on her (**a**) screening mammogram and persisted on (**b**) additional diagnostic spot compression views (arrows). The area was palpable by the patient and the clinician. The focal asymmetry had developed since her prior available mammogram performed 4 years earlier (**c**). Breast ultrasound (**d**) shows a corresponding irregular hypoechoic mass with associated posterior acoustic shadowing (arrow). Breast MRI sagittal post-contrast T1-weighted delayed images (**e**) and axial post-contrast T1-weighted images (**f**) show irregular enhancing masses (arrows). Corresponding T2 hyperintense cystic components with slit-like spaces (arrow) are identified (**g**), which favors PASH when present. Ultrasound-guided and MRI-guided biopsies were performed in this case and showed PASH. The area remained mammographically stable for over 4 years

Mammographically, PASH usually presents as an oval or round, noncalcified mass with circumscribed margins ranging from 0.3 to 11 cm (Fig. 11a, c) [43]. In some cases, it can present as a focal asymmetry (Fig. 12a–c). On sonography, it has a more variable appearance, from the more common and benign-appearing oval, circumscribed hypoechoic mass (Fig. 11b, d) to the irregular, mixed echogenicity mass (Fig. 12d) [43]. On MRI, PASH usually presents as a circumscribed mass resembling a fibroadenoma, but it can also present as non-mass enhancement in a focal or segmental distribution (Fig. 12e, f) [42, 43, 48]. The enhancement usually follows a progressive kinetics pattern. Presence of T2-hyperintense slit-like spaces with cystic components favors the PASH diagnosis favors the diagnosis of PASH (Fig. 12g).

Although it can coexist with malignant lesions [49, 50], PASH itself is a benign entity without an increased risk for malignancy [51]. To date, only a single case of PASH with malignant transformation has been reported [52]. In cases where PASH is identified as an incidental finding on pathology, no additional intervention is necessary, and clinical and imaging follow-up is recommended [53]. When PASH is identified as a mass on imaging or is the targeted lesion on biopsy, then surgical excision can be considered for larger lesions (> 2 cm) and for women with an increased risk of developing breast cancer or strong family history [43, 54, 55]. In the presence of an enlarging mass, surgical excision is recommended [45, 53]. Recurrence after excision of PASH has been reported at rates from 5 to 22% [43, 48].

### Tubular adenoma

Tubular adenoma is a rare benign tumor of the breast representing 0.13%-2.9% of all benign breast neoplasms [56–58]. It is more frequently seen in younger women of childbearing age. Although tubular adenoma and fibroadenoma are both epithelial tumors, a tubular adenoma is histologically distinguished from a fibroadenoma by its tightly packed tubular or acinar epithelium and sparse connective tissue. Conversely, a fibroadenoma has an extensive connective tissue component. Despite this, the cytologic features of tubular adenoma may still resemble those of a fibroadenoma. Therefore, histopathology remains key for confirmation of this diagnosis. Like fibroadenomas, tubular adenomas are not known to increase the risk of cancer, and in contrast to fibroadenomas, are not associated with pregnancy or oral contraceptive use [59, 60].

**Fig. 13:**
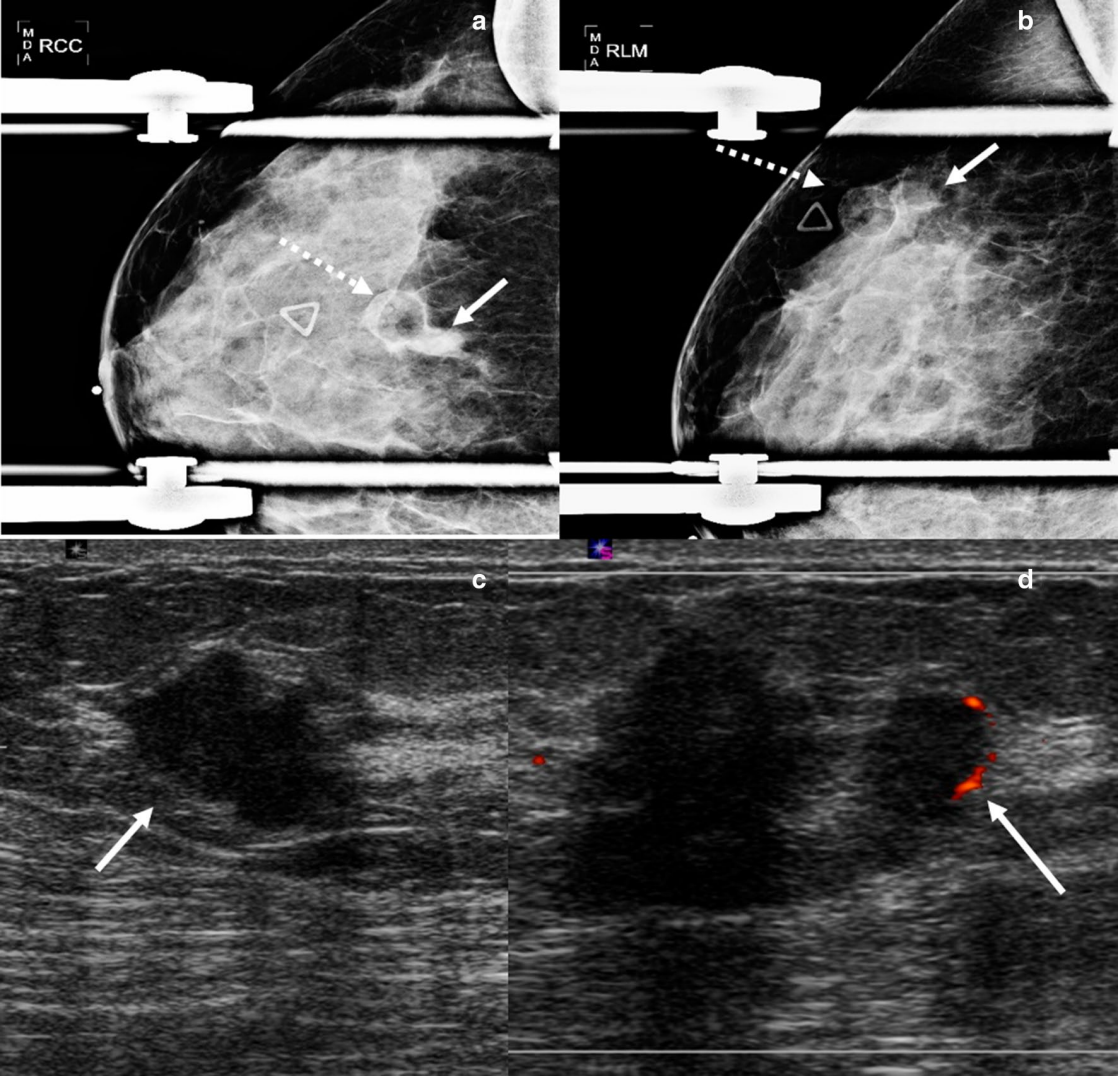
Tubular adenoma. A 36-year-old woman presented with a palpable breast mass. Spot cradiocaudal and lateral mammogram (**a**, **b**) shows a high-density irregular mass (solid arrow), adjacent to an incidental oil cyst (dotted arrow). Grayscale (**c**) and power Doppler ultrasound (**d**) images show an irregular hypoechoic mass with increased vascularity (arrows). Core needle biopsy showed tubular adenoma. The mass remained stable sonographically for 30 months and mammographically for 9 years

Both fibroadenomas and tubular adenomas present as circumscribed masses and are occasionally palpable. Mammographically, tubular adenomas have been associated with tightly grouped microcalcifications, which can be suspicious in morphology and may warrant biopsy [61]. Sonographically, tubular adenomas usually present as oval parallel masses with circumscribed margins, similar to fibroadenomas; however, tubular adenomas can also present as irregular hypoechoic masses (Fig. 13) [61]. Management should be determined by the clinical examination, imaging studies, and core biopsy results [62]. In many cases, as with a growing breast mass, surgical excision should be considered to obtain a definitive diagnosis [56].

### Desmoid fibromatosis

Desmoid fibromatosis is a rare benign mesenchymal tumor characterized by the proliferation of fibroblasts and myofibroblasts, accounting for 0.2% of all breast tumors [63]. Development of desmoid fibromatosis is associated with trauma, surgery, and Gardner syndrome [63, 64]. Despite its benign nature, it has a tendency for local recurrence [65]. Less than 10% of desmoid tumors are found in the breast; more common locations include the abdominal wall, retroperitoneum, and the mesentery. The clinical course of desmoid fibromatosis varies between patients.

**Fig. 14:**
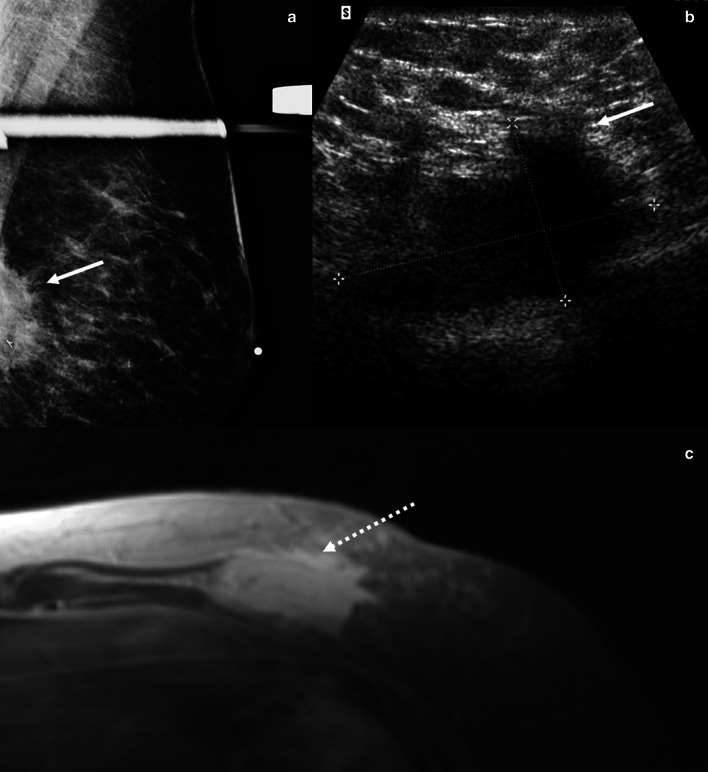
Desmoid fibromatosis. A 47-year-old woman presented with a palpable mass. Lateral spot compression mammogram (**a**) shows an irregular, high-density mass with spiculated margins adjacent to the chest wall (arrow). Grayscale ultrasound (**b**) shows a corresponding irregular, hypoechoic anti-parallel mass with indistinct margins. T1-weighted post-contrast fat-saturated MRI of the chest (**c**) shows invasion of the pectoralis major muscle by this irregular enahancing mass (dotted arrow). Core needle biopsy showed desmoid fibromatosis. The patient underwent surgical excision

The imaging features of desmoid fibromatosis are often indistinguishable from those of malignancy. Mammographically, desmoid fibromatosis presents as an irregular, high-density mass with spiculated margins (Fig. 14) [66]. On sonography, a hypoechoic irregular mass with posterior acoustic shadowing is usually seen [67]. MRI usually shows a heterogeneously enhancing mass, often with a progressive enhancement pattern. MRI is the modality of choice to assess extent of disease and to evaluate for chest wall involvement [68, 69].

The standard treatment for desmoid fibromatosis is surgical excision with wide margins, particularly in cases of tumor growth or progressive disease [70–75]. Additional treatment options include non-steroidal anti-inflammatory drugs, chemotherapeutic agents, hormonal therapy, and radiation treatment [71, 72]. Recently published studies have shown no significant difference in event-free survival between asymptomatic patients who underwent surgery and cases managed non-surgically, thereby suggesting that conservative management should precede surgical management [71, 72]. The Desmoid Working Group and European Society for Medical Oncology have provided additional guidance, adding the possibility of active surveillance to the above-stated treatment choices with the supervision of a multidisciplinary team experienced in desmoid fibromatosis tumors. In the presence of tumor growth or progressive disease, surgery remains the gold standard [69, 72–74].

### Granular cell tumor

**Fig. 15:**
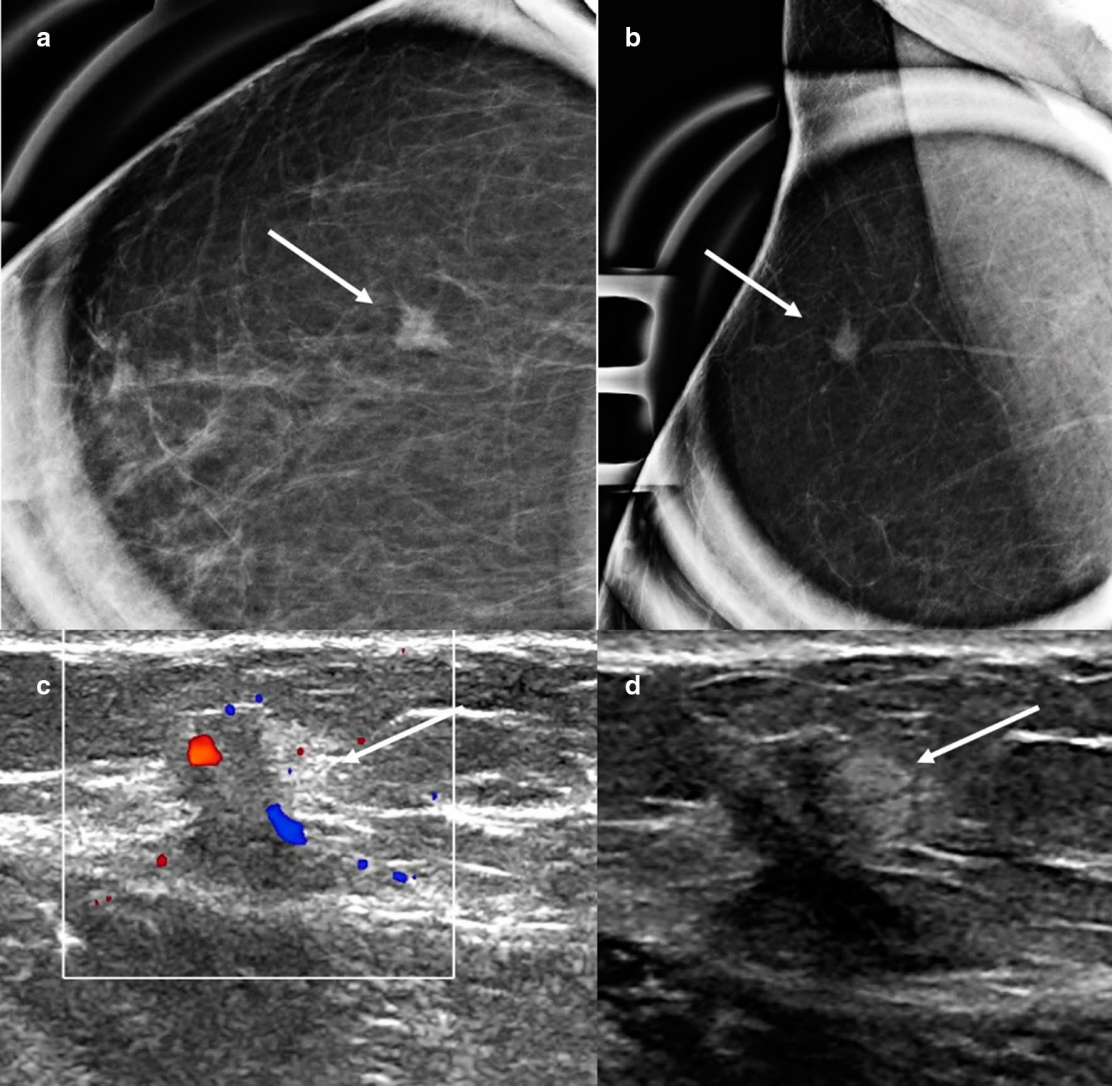
Granular cell tumor. A 65-year-old woman presented for further evaluation of a focal asymmetry detected on screening mammopgrahy. Craniocaudal and lateral spot compression views (**a**, **b**) show a small irregular mass with spiculated margins (arrows). Color Doppler (**c**) and grayscale (**d**) ultrasound shows a corresponding irregular mass with internal vascularity (arrows). Core needle biopsy yielded granular cell tumor which was treated with surgical excision

Granular cell tumor is an extremely rare tumor of neural origin. It is more commonly found in the head and neck and chest wall regions, with only 4%-6% of cases located in the breast [76, 77]. Granular cell tumor has a predilection for premenopausal and African American women and is preferentially found in the upper inner quadrants of the breast. The clinical and imaging features of granular cell tumor are indistinguishable from those of breast cancer. On physical examination, it can present as a fixed, painless, palpable mass with associated skin dimpling. Mammographically, granular cell tumors can present as a mass with indistinct or spiculated margins (Fig. 15) [42]. On sonography, a hypoechoic mass with irregular or circumscribed margins is usually seen. On MRI, a mass with persistent or washout kinetics is usually seen [78]. Malignant granular cell tumors occur in 1% of these cases [77]. The most common sites of metastasis are the lymph nodes and lungs. PET can differentiate between benign and malignant granular cell tumors, with the benign tumors showing a lower standardized uptake value than the cutoff value of 2.5 [79]. Biopsy is indicated for diagnosis. Although granular cell tumors are benign, wide local surgical excision is indicated owing to their locally infiltrative nature [75, 80].

## Conclusion

Awareness of multiple malignancy mimickers, rare and common, is vital to clinical practice and plays a key role in radiologic-pathologic concordance, ensuring appropriate clinical management.

## Data Availability

Not applicable.

## Abbreviation

PASH: Pseudoangiomatous stromal hyperplasia
